# Evaluation of influenza vaccine effectiveness and description of circulating strains in outpatient settings in South Africa, 2014

**DOI:** 10.1111/irv.12314

**Published:** 2015-06-09

**Authors:** Johanna M McAnerney, Florette Treurnicht, Sibongile Walaza, Adam L Cohen, Stefano Tempia, Senzo Mtshali, Amelia Buys, Lucille Blumberg, Cheryl Cohen

**Affiliations:** a National Institute for Communicable Diseases (NICD), National Health laboratory services (NHLS)Johannesburg, South Africa; b School of Public Health, Faculty of Health Sciences, University of the WitwatersrandJohannesburg, South Africa; c U.S. Centers for Disease Control and PreventionAtlanta, GA, USA; d U.S. Centers for Disease Control and Prevention – South AfricaPretoria, South Africa

**Keywords:** Effectiveness, influenza, vaccine

## Abstract

The effectiveness of the trivalent seasonal influenza vaccine during the 2014 season in South Africa was assessed using a test-negative case–control study design including 472 cases and 362 controls. Influenza A(H3N2) was the dominant strain circulating. The overall vaccine effectiveness estimate, adjusted for age and underlying conditions, was 43·1% (95% CI: −26·8–74·5). 2014 H3N2 viruses from South Africa were mainly in sublineage 3C.3 with accumulation of amino acid changes that differentiate them from the vaccine strain in 3C.1.

## Introduction

In South Africa, annual influenza vaccination is recommended for individuals at increased risk or healthy individuals wishing to reduce their risk of contracting influenza.^1^ A limited number of doses are available free of charge in the public sector for high-risk groups, which include young children, the elderly, pregnant or postpartum (within 2 weeks of delivery) women, and persons of any age with underlying medical conditions (such as heart disease, lung disease and HIV infection). In addition, anyone wishing to protect themselves against influenza can purchase vaccine in the private sector. The Southern Hemisphere trivalent influenza vaccine (TIV) for 2014 contained an A/California/7/2009 (H1N1)-like virus; an A/Texas/50/2012 (H3N2)-like virus; and a B/Massachusetts/2/2012-like virus, similar to recommendations for the 2014/15 Northern Hemisphere season. During the 2013/14 Northern Hemisphere influenza season, vaccine effectiveness (VE) was shown to be 61% in the USA,^2^ and 74% in Canada where influenza A(H1N1)pdm09 predominated.^3^ New Zealand, in the Southern Hemisphere, showed an interim 59% VE against hospitalisation and 74% effectiveness against medically attended acute respiratory infection in the 2014 predominantly A(H1N1)pdm09 season.^4^ However, influenza vaccine was not shown to be effective in Spain in 2013–2014 season where A(H3N2) strains predominated.^5^ Despite relatively low coverage (≈3·5%) among patients attending general practitioners in South Africa, TIV has been shown to be effective against influenza-associated medically attended acute respiratory illness using a test-negative case–control study design in years when good vaccine strain match was reported (2010, 2011, 2013).^6^ We aimed to estimate TIV effectiveness against laboratory-confirmed medically attended influenza illness for the 2014 influenza season in South Africa and characterise circulating strains.

## Methods

The Viral Watch programme is an influenza sentinel surveillance programme.^7^ In 2014, 101 outpatient practitioners at 65 practices in eight of the nine provinces of South Africa participated in the programme. Patients presenting with influenza-like illness (ILI) to participating practitioners and testing influenza virus positive were defined as cases, whereas those who tested negative were controls. ILI was defined as acute respiratory illness with a measured temperature of ≥38°C or a history of fever, and cough, with onset within the past 10 days. Throat and/or nasal swabs were taken from a maximum of five patients per week as part of routine diagnostic investigations for which informed written consent was not required. The choice of patients sampled was left to the practitioner's discretion.

Specimens were tested using multiplex reverse transcription real-time polymerase chain reaction (rRT-PCR) assays for influenza A and B. Influenza A-positive specimens were further subtyped by rRT-PCR.^8^–^10^ The haemagglutinin (HA) genes H3N2 strains from a convenience sample of participants from the beginning, mid- and end season (*n* = 34) were amplified and sequenced, and phylogenetic analysis using maximum-likelihood tree construction and pairwise distances (p-distance) was performed for the HA1 region (surface domain) using mega5.2.^11^ The number of N-linked glycosylation sites was determined as described by Zhang *et al*.^12^ Influenza virus isolation in Madin–Darby Canine kidney cells was performed for influenza H3N2 virus-positive samples with a crossing point threshold ≤30 (*n* = 30), and antigenic reactivity of isolates (*n* = 26) was determined by haemagglutination inhibition (HAI) assay using turkey red blood cells as indicator and vaccine strain-specific antisera produced in rabbits (HAI typing kit 2014; A/Texas/50/2012 Lot no. KAS414-1 VIDRL, WHO Collaborating Centre, Melbourne, Australia).

Clinical, demographic and influenza vaccination data were collected from each patient at the time of specimen collection. Patients aged ≥6 months meeting the ILI case definition with available influenza vaccine history were included in the VE analysis. Vaccine history was self-reported or from provider records, where available, and it was not recorded whether children <9 years had received two doses. Patients who had received the current season influenza vaccine >14 days prior to the onset of illness were considered vaccinated. Patients who had received influenza vaccine ≤14 days prior to onset of symptoms were excluded.

The start of the influenza season was defined as two consecutive weekly influenza detection rates of ≥10%, and the end as when the detection rate dropped below 10% for two consecutive weeks, or <10 specimens per week were received.^7^ The season was divided into three equal parts as follows: early (weeks 21–26); mid (weeks 27–32); late (weeks 28–37). Finer time units were not used due to small sample size. Only specimens collected during the season were included in the VE analysis. Multivariate logistic regression was used to adjust VE estimates by age, pre-existing underlying medical conditions putting the patient at risk of complications of influenza, and seasonality (first, mid or last third of the season). Vaccine effectiveness was calculated as 1-odds ratio for laboratory-confirmed influenza in vaccinated and unvaccinated patients. All analyses were conducted using stata version 12.1 (Statacorp LP, College Station, TX, USA.)

## Results

**Figure 1:**
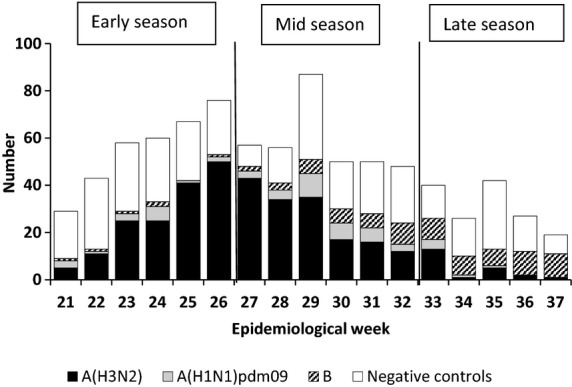
Test-negative controls and laboratory-confirmed cases by week and virus subtype: Viral Watch programme, South Africa, 19 May – 14 September 2014.

The 2014 influenza season in South Africa started in week 21 (week ending 25 May) and ended in week 37 (week ending 14 September) during which time 857 individuals were enrolled and tested and of whom 834 (97·3%) were eligible for the VE analysis. Among individuals included, the overall influenza detection rate was 56·6% (472/834). The season was dominated initially by influenza A(H3N2) which accounted for 336/472 (71·2%) of the total influenza subtypes detected; influenza B dominated in the last third of the season, accounting for 44/71 (62·0%) of influenza detections compared with 6/179 (3·4%) in the first third of the season. Influenza A(H1N1)pdm09 co-circulated throughout the season in low numbers with a total of 54/472 (11·4%) detections (Figure1).

Nearly half (354, 42·2%) were patients aged 20–44 years, and 437 (52·4%) patients were female. Mid-season specimens accounted for 41·7% (348) of the total, although this proportion was higher [222 (47·0%)] for cases. The majority of specimens [743 (89·0%)] were collected within 3 days of symptom onset. Pre-existing underlying medical conditions, such as chronic pulmonary and cardiac disease, immunosuppression (including HIV), metabolic disorders, pregnancy and morbid obesity defined as a body mass index of ≥40, were reported in 149 (17·9%) patients (Table1).

**Table 1:** Characteristics of cases (influenza test positive) and controls (influenza test negative) in the Viral Watch Programme, South Africa, 2014

Variable	Cases *N *= 472	Controls *N* = 362	Total *N* = 834	*P*
	*n* (%)	*n* (%)	*n* (%)	
Age group				
Median years	34	32	33	0·06
<20 years	131 (27·8)	107 (29·6)	238 (28·5)	
20 – 44 years	189 (40·0)	165 (45·6)	354 (42·4)	
≥45 years*	152 (32·2)	90 (24·8)	243 (29·1)	
Sex				
Male	230 (48·7)	165 (45·4)	395 (47·4)	0·41
Female	242 (51·3)	192 (53·0)	437 (52·4)	
Unknown		2 (0·6)	2 (0·2)	
Seasonality				
Early (weeks 21–26)	179 (37·9)	153 (42·3)	332 (39·1)	<0·01
Mid (weeks 27–32)	222 (47·0)	126 (34·8)	348 (41·7)	
Late (weeks 33–37)	71 (15·0)	83 (2)	154 (18·5)	
Region				
Central Plateau**	265 (56·1)	175 (48·3)	440 (52·8)	0·03
North East Subtropical***	60 (12·7)	66 (18·2)	126 (15·1)	
Southern coastal belt†	147 (31·2)	121 (33·5)	268 (32·1)	
Underlying condition††				
None	379 (80·3)	304 (84·0)	683 (81·9)	0·37
Yes	92 (19·5)	57 (15·7)	149 (17·8)	
Unknown	1 (0·2)	1 (0·3)	2 (0·3)	
Interval between onset and sampling (days)				
0–3 days	433 (91·7)	310 (85·6)	743 (89·1)	<0·01
4–10 days	39 (8·3)	52 (14·4)	91 (10·9)	

* We were unable to stratify by the age group ≥65 years due to small numbers of patients in this age group.

** Free State, Gauteng, Northern Cape and North West Provinces.

*** Mpumalanga and Limpopo Provinces.

† Eastern Cape and Western Cape Provinces.

†† Chronic pulmonary and cardiac disease, chronic renal disease, diabetes and similar metabolic disorders, immunosuppression, morbid obesity (BMI ≥ 40), and pregnancy and post-parturition.

Overall, the influenza vaccine coverage was 3·0% in cases and 3·6% in controls. Coverage in patients with underlying conditions was 6·5% in cases and 3·5% in controls and in those aged ≥45 years was 5·9% in cases and 5·6% in controls (Table2). Of the 27 patients vaccinated, 14 were positive for influenza A(H3N2), of whom 10 belonged to groups at high risk for complications of influenza including young children, the elderly, and persons with underlying conditions.

**Table 2:** Vaccine receipt and vaccine effectiveness (VE) estimates by the presence of underlying medical conditions (UMC), age group and timing within season, Viral Watch Programme, South Africa, 2014

	Vaccine coverage			Percentage crude VE (95%CI)
	Cases	Controls	Total	
	*n*/*N* (%)	*n*/*N* (%)	*n*/*N* (%)	
Total	14/472 (3·0)	13/362 (3·6)	27/834 (3·2)	17·9 (−76·8, 61·9)
UMC	6/92 (6·5)	2/57 (3·5)	8/149 (5·4)	−91·9 (−884·8, 62·6)
No UMC	8/379 (2·1)	11/307 (3·6)	19/683 (2·8)	42·6 (−44·6, 77·2)
<20 years	1/131 (0·8)	2/107 (1·9)	3/238 (1·3)	59·6 (−351·3, 96·4)
20–44 years	4/189 (2·1)	6/165 (3·6)	10/354 (2·8)	42·7 (−106·7, 84·1)
≥45 years	9/152 (5·9)	5/90 (5·6)	14/242 (5·8)	−7 (−229·8, 65·3)
Early season (weeks 21–26)	1/126 (0·8)	1/130 (0·8)	2/256 (0·8)	−3·2 (−1568, 36·2)
Mid-season (weeks 27–32)	12/223 (5·4)	7/103 (6·8)	19/326 (5·8)	22·0 (−104·3, 70·2)
Late season (weeks 33–37)	1/123 (0·8)	5/129 (3·9)	6/252 (2·4)	79·7 (−76·5, 76·6)

The overall VE estimate, adjusted for age, underlying conditions and seasonality, was 43·1% (95% CI: −26·8–74·5) against any influenza virus type. The adjusted overall VE estimate against influenza A(H3N2) was −18·4% (95% CI: −171·5–48·4). We were not powered to obtain estimates stratified by influenza strain, age group or the presence of underlying illness.

**Figure 2:**
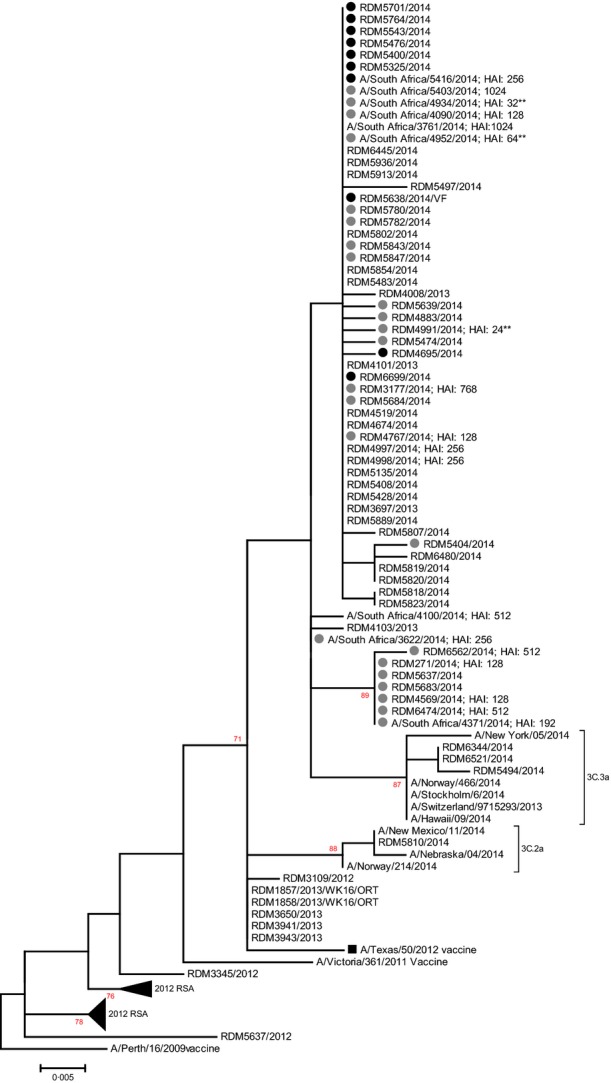
Maximum-likelihood tree of the A(H3N2) HA1 region (850 bp) including strains from the South African 2014 influenza season. The 2014 VW strains are indicated by circles (black = vaccine breakthrough strains). The 2014 Southern Hemisphere (SH) vaccine strain A/Texas/50/2012 is indicated by a black square. The emerging sub-subgroups 3C.2a and 3C.3a with low reactivity to the current vaccine are indicated. Haemagglutination inhibition titres are indicated next to strains (** low reactors).

**Figure 3:**
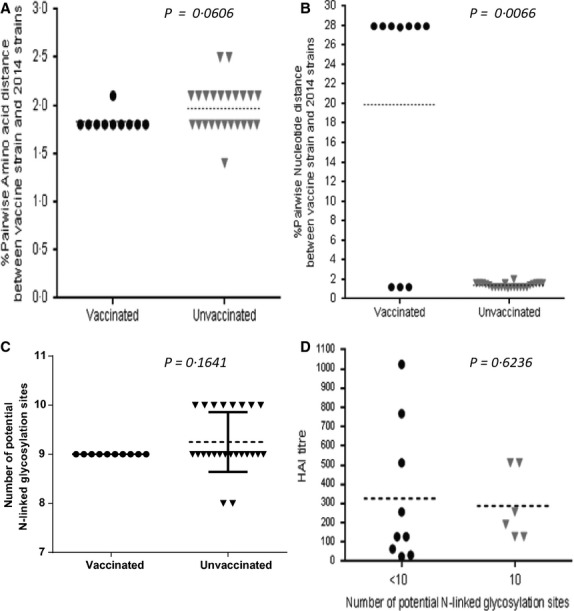
Graphic presentation of (A) pairwise amino acid distance and (B) pairwise nucleotide distance to the 2014 vaccine strain, (C) the number of potential N-linked glycosylation sites in H3N2 strains from vaccinated and unvaccinated VW participants as well as (D) determining the effect of number of N-linked glycosylation sites on antigenic relatedness to the vaccine strain using haemagglutination inhibition titres.

The 2014 influenza A(H3N2) viruses from South Africa (*n* = 34) were mainly in sublineage 3C.3 with accumulation of amino acid changes that differentiate them from the vaccine strain in 3C.1. (Figure2). Vaccine breakthrough infection strains (10/34) were characterised by S124N, R142G and N145S mutations in epitope A and N128A and S198P mutations in epitope B which is the same as for two-thirds (16/24) of viruses from unvaccinated participants. Amino acid p-distance between the 2014 vaccine strain was increased in unvaccinated participants compared with vaccinated in 2014 although not significantly (Mann–Whitney two-tailed=0·061) (Figure3A). However, a significant increase in nucleotide p-distance (Figure3B) was observed in strains from vaccinated participants (Mann–Whitney two-tailed = 0·007). Viruses from unvaccinated participants, when compared to those from vaccinees, had an increased number of potential N-linked glycosylation sites (Figure3C) similar to the vaccine strain, although not significant (Mann–Whitney two-tailed = 0·164). No difference was observed in antigenic reactivity (Figure3D) to vaccine strain antisera between strains with <10 and those with 10 N-linked glycosylation sites (Mann–Whitney two-tailed = 0·624).

## Discussion

Our point estimates for the Southern Hemisphere vaccine are lower than (but not statistically different from) the interim estimates reported from New Zealand where the 2014 season was dominated by influenza A(H1N1)pdm09.^4^ This is the second season, since the 2009 pandemic, that influenza A(H3N2) was the dominant subtype in South Africa, and in both seasons, we were unable to demonstrate significant VE, with a 38·4% point estimate in 2012.^13^ It has been reported elsewhere that VE against influenza A(H3N2) is often lower than that against A(H1N1).^10^ Our VE results have occurred in the context of an antigenic drift of the A(H3N2) viruses which circulated in South Africa. Antibody binding sites for neutralising antibodies induced by the 2014 vaccine strain may have been masked or changed on haemaglutinin spikes of viruses associated with vaccine breakthrough infections which had lost an N-linked glycosylation site at position 124. As expected, the lowest HAI titres were seen for viruses with fewer glycosylation sites. The increase in nucleotide changes in vaccine breakthrough infection strains that are synonymous do not change the HA1 protein and antigenicity but may negatively affect viral RNA signalling or packaging which could decrease viral replication and survival of these variants.^14^–^16^ These findings highlight the importance of the continuous monitoring of genetic changes in the haemaglutinin domain during seasonal epidemics.

The Northern Hemisphere influenza vaccine composition for the 2014–2015 season was the same as the vaccine composition used during the Southern Hemisphere 2014 influenza season.^13^ During the 2014/15 season, the United Kingdom experienced circulation of A(H3N2) and mid-season estimates showed an adjusted VE of 3·4% against primary care consultation and −2·3% against laboratory-confirmed influenza.^17^ Influenza A(H3N2) circulating in Europe was antigenically and/or genetically different from the vaccine strain.^18^ The 2014/15 season in North America has also been dominated by influenza A(H3N2). In the United States, early VE estimates indicated that the influenza vaccine provided limited protection.^19^ In Canada, interim-unmatched VE estimates adjusted for age and medical comorbidities were determined to be −16·8% overall, and −22·0% for laboratory-confirmed influenza A(H3N2).^20^ Viruses clustered with phylogenetic clade 3C.2a considered genetically and antigenically different from the vaccine strain. Interim-adjusted VE against A(H3N2) was −8%.^21^ During spring 2014, drifted influenza A(H3N2) viruses, subgroup 3C.3a, were detected in Finland. Another subgroup, 3C.2a emerged in the 2014/15 season and has predominated. Antibody response data suggested reduced protection against vaccine strain.^22^ Both these subgroups were detected in South Africa during the 2014 season Figure2.

There are several limitations to our study which was not specifically designed to assess VE and, given the available data, we were not powered to statistically assess significance of VE estimates lower than 80%. In addition, the low vaccine coverage affected the ability to statistically estimate significance of VE among smaller subgroups such as the elderly (>65 years of age). Influenza vaccine status and underlying conditions were self-reported by some patients to the practitioner which could have led to misclassification and residual confounding. In addition, a higher proportion of controls had a longer period between onset and collection which can introduce misclassification bias through false negatives. Our study population mainly included individuals accessing private health care through general practitioners and may not be representative of the public sector where vaccine coverage is lower.^6^ The gold standard for determining antigenic profiles of influenza viruses is the HAI assay using ferret antisera and number of isolates obtained for HAI assays was small.

## Conclusions

Based on our data, the 2014 TIV had a <50% point estimate for effectiveness against outpatient ILI in South Africa. Previous estimates from South Africa using the same methodology have found TIV to be effective in this population.

## Conflict of interest

The authors do not declare any conflict of interest.

## Role of the funding source and financial disclosure

This study received funding from the NICD/NHLS and was supported in part by funds from the United States Centers for Disease Control and Prevention (CDC), Atlanta, Georgia Preparedness and Response to Avian and Pandemic Influenza in South Africa (Cooperative Agreement Number: U51/IP000155-04). The contents are solely the responsibility of the authors and do not necessarily represent the official views of the CDC. The funders had no role in study design, implementation, manuscript writing or the decision to submit for publication. The corresponding author had full access to all the data in the study and takes final responsibility for the decision to submit for publication.

## Ethics statement

The protocol was approved by the Research Ethics Committees of the University of the Witwatersrand (reference number M060449). The Centers for Disease Control and Prevention's Institutional Review Board relied on the ethical approval from the University of the Witwatersrand. Individual informed consent was not required as participants were tested for influenza for diagnostic purposes and as part of surveillance.
